# Dataset for gait analysis and assessment of fall risk for older adults

**DOI:** 10.1016/j.dib.2020.106550

**Published:** 2020-11-21

**Authors:** Pablo E. Caicedo, Carlos F. Rengifo, Luis E. Rodriguez, Wilson A. Sierra, Maria C. Gómez

**Affiliations:** a Faculty of Engineering, Corporación Universitaria Autónoma del Cauca, Colombia; b Department of Electronics, Instrumentation and Control, Universidad del Cauca, Colombia; c Biomedical Engineering Program, Escuela Colombiana de Ingeniería Julio Garavito, Colombia; d Clínica Universidad de La Sabana, Colombia

**Keywords:** Gait analysis, Fall risk assessment, Performance oriented mobility assessment, POMA, Short physical performance battery, SPPB, Gait parameters, Motor function test

## Abstract

This article presents a five-part dataset for human gait analysis in a healthy older adult population (37 women and seven men; age 69.98 ±8.57 years, body mass index 27.71 ±4.57). Part 1 compromises demographic data for the 44 participants, along with the results of the Short Physical Performance Battery (SPPB) motor function test and the Mini-Mental State Examination (MMSE). We used the MMSE to ensure the participants could understand the experimental protocol instructions. Part 2 presents the anthropometric measurements of the participants. Part 3 shows the results for each item of the Gait component of the Performance Oriented Mobility Assessment (POMA-G). Part 4 contains five files per participant, storing motion-capture data for the lower limb in the Coordinate 3D (C3D) format. These files were generated using a Vicon motion analysis system, consisting of 24 reflective markers and seven cameras (Vantage V5) sampled at 100 Hz. Part 5 contains 26 gait parameters for each C3D file obtained using Nexus 2.9.3. The dataset is available in a Mendeley repository (Reserved DOI: 10.17632/xgw6bg3g8h.1).

## Specifications Table

**Table:** 

Subject	Biomechanics
Specific subject area	Human gait analysis is the systematic study of how locomotion patterns vary between people according to specific characteristics such as age, anthropometry, pathologies, or walking conditions.
Type of data	Table Coordinate 3D (C3D) files
How data were acquired	Kinematic data were collected using a Vicon motion analysis system at 100 Hz. A physiotherapist administered SPPB [1], POMA-G [2], and MMSE [3] to 44 participants. The spatial and temporal gait parameters were calculated using the Nexus 2.9.3 software.
Data format	Raw and analyzed.
Parameters for data collection	An engineer with six years of experience in gait analysis placed 24 markers on each participant according to Conventional Gait Model version 2.3 (CGM2.3) [4, 5]. The 24 markers comprised ten markers on each leg and four around the pelvis.
Description of data collection	The dataset contains demographic and anthropometric data, the global test scores for the SPPB and MMSE, the assessment of each item on the POMA-G when evaluated by two physiotherapists, five C3D files per participant, and 26 parameters for each participant on the C3D file.
Data source location	Escuela Colombiana de Ingeniería Julio Garavito. Bogotá D.C./Cundinamarca/Colombia 4.7827° N, 74.0426° W
Data accessibility	Reserved DOI:10.17632/xgw6bg3g8h.1 https://data.mendeley.com/datasets/xgw6bg3g8h/1

## Value of the Data

• The dataset is intended to train machine-learning models to predict analytical outcomes with the SPPB and POMA-G from the Cartesian position of the reflective markers. The data set can also be used to compare fall risk assessments based on the SPPB and POMA-G versus techniques such as Lyapunov exponents [6, 7] Floquet multipliers [8], and recurrence quantification analysis [9].
• Researchers and clinicians who use data-oriented algorithms to assess fall risk will benefit from this dataset because they can use the Cartesian position of the markers as inputs for their algorithms. Then, they can compare the resulting evaluation with screening tools accepted in their clinical environments.
• The dataset should serve as a basis to validate new fall risk assessment algorithms that complement motor function tests, such as SPPB and POMA. This use of the dataset as a complementary approach is effective because in these motor function tests, the resulting scores can be influenced by the test administrator's experience and level of concentration.

## 1 Data Description

File *01_demography_sppb_mmse.xlsx* contains each participant's sex, age, mass, height, body mass index, number of self-reported falls in the last month, and the results of SPPB and MMSE.

**Fig. 1:**
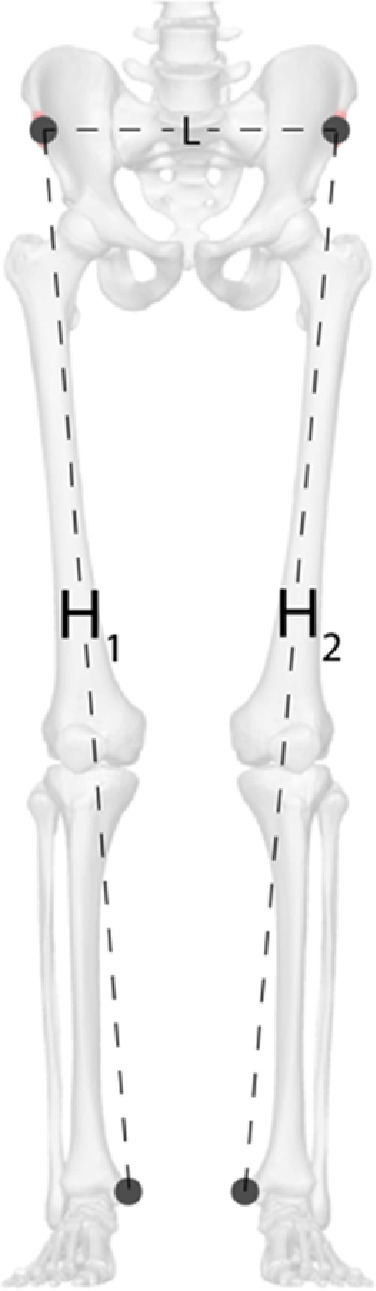
Anthropometric distances for each participant. Adapted from the original available in https://upload.wikimedia.org/wikipedia/commons/6/67/ASIS_01_anterior_view.png.

File *02_anthropometry.xlsx* contains the linear distance between right and left anterior superior iliac spine (L), distance between right anterior superior iliac spine and right medial malleolus (H_1_), distance between left anterior superior iliac spine and left medial malleolus (H_1_), left knee width, right knee width, left ankle width, and right ankle width for each participant. Fig. 1 shows the first three distances.

File *03_poma_test.xlsx* contains some participant's results for the seven POMA-G test items. Each participant was filmed by three cameras while walking in a straight line through a room. Two physiotherapists independently evaluated the videos. One physiotherapist was an expert, who has administered the test daily for the past five years. The other physiotherapist was familiar with the test but has administered it once a year. Each physiotherapist-rater evaluated the videos twice with a difference of one week. These data are intended to calculate intra and interrater agreement. The missing results are due to unexpected failures in video cameras.

**Fig. 2:**
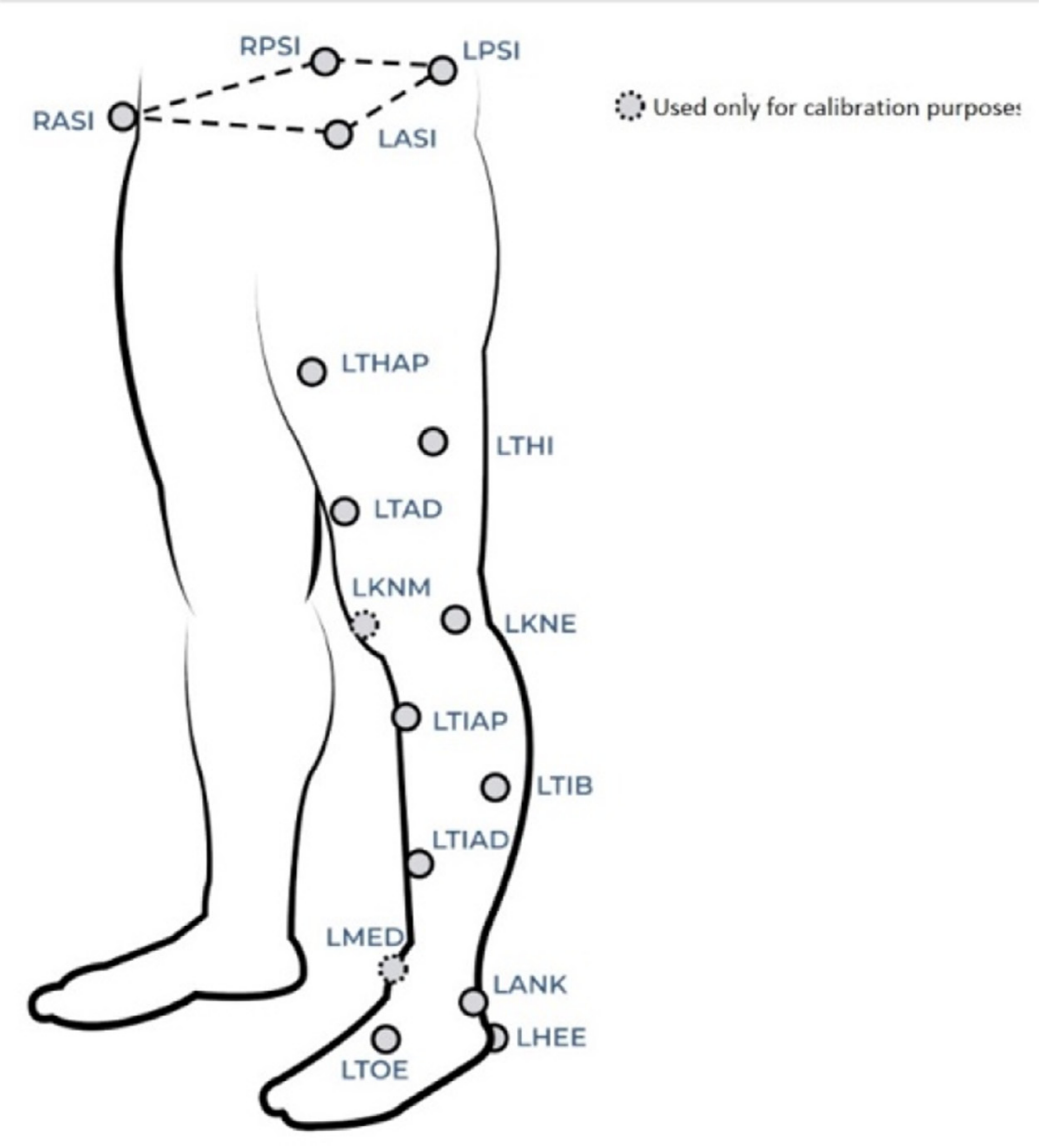
Reflective markers located according to CGM2.3. Image from https://pycgm2.github.io/pages/CGM23-Overview.html.

Folder *04_C3D_files* contains five C3D files per each participant. Each participant walked five times using 24 reflective markers, ten at each leg and four around the hip (Fig. 2). The markers were located according to CGM2.3 [4, 5].

File *05_gait_parameters.xlsx* contains 26 gait spatiotemporal gait parameters for each participant. Each parameter is described at this link. https://www.vicon.com/support/faqs/?q=how-does-nexus-plug-in-gait-and-polygon-calculate-gait-cycle-parameters-spatial-and-temporal.

## 2 Experimental Design, Materials and Methods

### 2.1 Materials

The optical motion capture system comprised seven cameras (Vantage V5) sampled at 100 Hz and distributed around a rectangular area 15 m long and 6 m wide. Each camera was mounted on a tripod 1.90 m above the floor. The gait parameters were calculated according to the CGM2.3 by Nexus movement analysis software, version 2.9.3. During system calibration, which was repeated every day during the ten days required to capture the data for the 44 participants, the Vicon software reported an accuracy better than 0.3 mm.

**Fig. 3:**
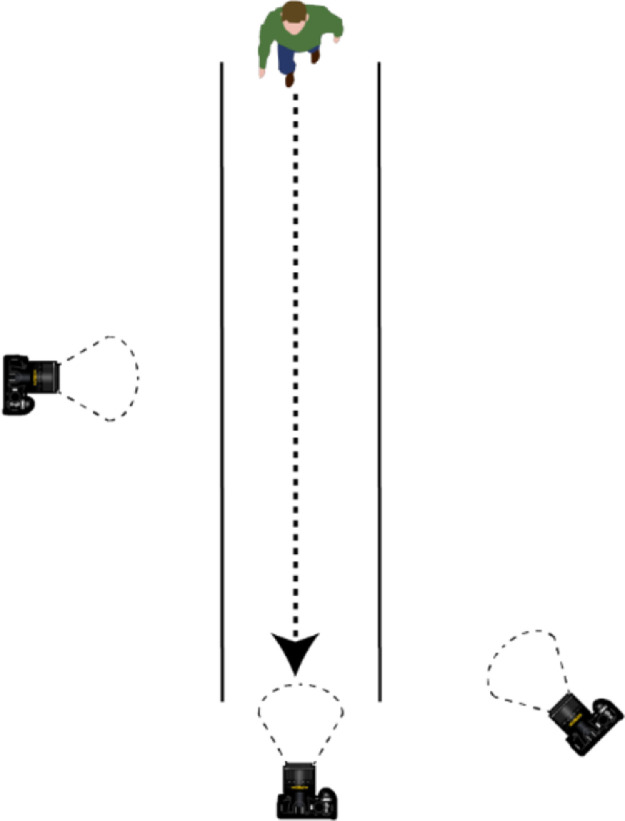
Position of the cameras used to film the participants during the optoelectronic motion-capture trials.

Also, three RGB cameras (Thieye T4, Thieye T5 y Thieye T5 edge) were distributed as indicated in Fig. 3 to film the participants while they were walking and wearing reflective markers. Thus, both the Vicon and the RGB cameras recorded the participant's movement simultaneously.

### 2.2 Data collection protocol

This experimental protocol was used to obtain the dataset:

1. A physiotherapist asks the participant's sex, age, and the number of self-reported falls over the last month, along with the participant's mass and height to calculate body mass index.
2. A physiotherapist administers the MMSE.
3. A physiotherapist administers the SPPB.
4. An engineer identifies bony landmarks and measures inter anterior-superior-iliac-spine distance, pelvic depth, clinical leg length, knee diameter, and ankle diameter. These measurements are required to calculate joint angles from the Cartesian positions of the markers.
5. The participant is asked to remain immobile to estimate the joint's center of rotation using the CGM2.3 and the anthropometric parameters obtained from the previous step.
6. The participant is asked to walk ten times between two points at a distance of 12 m apart. For each gait, a C3D file and three videos are generated.

### 2.3 Data processing

Table 1 presents the data-processing steps carried out using Nexus.

**Table 1:** Data processing for data collection protocol Steps 5 and 6.

Data processing sequence	Step of the data collection protocol	
	5	6
Assign labels to the markers according to CGM2.3 (Fig. 2).	Yes	Yes
Eliminate false markers generated by unwanted reflections.	Yes	Yes
Fill the gaps in the trajectories of the four markers of the pelvis using the Nexus Rigid Body Fill option, which is used only when the relative positions between the markers are constant.	Yes	Yes
Apply the sequence of filling options: (i) Rigid Body Fill, (ii) Pattern Fill, and (iii) Spline Fill to the markers in thighs, shanks and feet.	Yes	Yes
Fit CGM2.3 to each participant using the tools: (i) Scale Subject, (ii) Static Skeleton Calibration – Markers Only, and (iii) CGM2.3 – Calibration.	Yes	No
Filter the resulting trajectory using the pipeline Filter Trajectories – Woltring.	No	Yes
Calculate the gait parameters using the pipeline Calculate Gait Cycle Parameters, which is a Vicon's implementation of CGM2.3.	No	Yes

The best five trials of each participant were included in the dataset. These trials have 0 unused markers and 0 markers unlabeled.

### 2.4 Fall risk assessment using POMA

Two physiotherapist used POMA-G to assess participants by watching three videos of each participant (Fig. 3). These videos were looped for one minute, and then the raters had two minutes to complete the seven POMA-G questions. This process was repeated for the videos of each participant. Then, one week later, the physiotherapist-raters used POMA-G again to assess the participants by watching the same videos; however, for this new rating session, the videos were presented in a different order than in the previous week.

## Ethics Statement

The experiments described in this work have been carried out following the Code of Ethics of the World Medical Association (Declaration of Helsinki) for experiments involving humans. Also, the participants gave their informed consent approved by the Ethics Committees of both, the Escuela Colombiana de Ingeniería and Clínica Universidad de la Sabana.

## Declaration of Competing Interest

The authors declare that they have no known competing financial interests or personal relationships which have, or could be perceived to have, influenced the work reported in this article.
